# How to survive (peri)menopause as a woman with a neurodevelopmental disorder

**DOI:** 10.1192/j.eurpsy.2026.11499

**Published:** 2026-07-31

**Authors:** A. Todzia-Kornaś

**Affiliations:** Department of Psychiatry, Combat Stress and Psychotraumatology, Military Institute of Medicine - National Research Institute, Warsaw, Poland

## Abstract

**Introduction:**

Perimenopause is characterized by dynamic fluctuations in ovarian hormones, particularly estrogen and progesterone, which exert modulatory effects on neurotransmitter systems implicated in cognition, mood regulation, and executive functioning.

**Objectives:**

In women with neurodevelopmental disorders such as attention-deficit/hyperactivity disorder (ADHD) and autism spectrum disorder (ASD), these hormonal transitions may interact with pre-existing neurobiological vulnerabilities, contributing to symptom exacerbation and functional decline.

**Methods:**

Despite increasing recognition of sex- and gender-specific presentations of ADHD and ASD, the impact of perimenopausal neuroendocrine changes remains largely underexplored. Preliminary evidence suggests that estrogen withdrawal may intensify deficits in dopaminergic and serotonergic signaling, potentially worsening attentional control, emotional regulation, and social cognition. Clinical reports further indicate heightened psychiatric comorbidity, treatment resistance, and decreased quality of life during this life stage.

**Results:**

This workshop will provide an overview of current evidence on the intersection of perimenopause and neurodevelopmental disorders, including neurobiological mechanisms, clinical phenomenology, and methodological limitations in existing research. We will discuss implications for future studies, but also the role of psychopharmacology, hormone replacement therapy, and multimodal interventions.

**Conclusions:**

By integrating neuroscientific insights with clinical observations, this workshop aims to delineate a research agenda and stimulate interdisciplinary dialogue on a neglected yet critical dimension of women’s mental health.

**Disclosure of Interest:**

None Declared

